# Evidence of Linezolid Resistance and Virulence Factors in *Enterococcus* spp. Isolates from Wild and Domestic Ruminants, Italy

**DOI:** 10.3390/antibiotics11020223

**Published:** 2022-02-10

**Authors:** Camilla Smoglica, Alberto Vergara, Simone Angelucci, Anna Rita Festino, Antonio Antonucci, Fulvio Marsilio, Cristina Esmeralda Di Francesco

**Affiliations:** 1 Faculty of Veterinary Medicine, University of Teramo, Loc. Piano D’Accio, 64100 Teramo, TE, Italy; avergara@unite.it (A.V.); simone.angelucci@parcomajella.it (S.A.); arfestino@unite.it (A.R.F.); fmarsilio@unite.it (F.M.); cedifrancesco@unite.it (C.E.D.F.); 2 Maiella National Park, Wildlife Research Center, Viale del Vivaio, 65023 Caramanico Terme, PE, Italy; antonio.antonucci@parcomajella.it

**Keywords:** wild animals, *Enterococcus*, linezolid, antimicrobial resistance, virulence factors, One Health

## Abstract

The aim of this study was to evaluate the resistance patterns against selected critically and highly important antibiotics (quinupristin/dalfopristin, vancomycin, and linezolid) in 48 *Enterococcus* isolates obtained from wild (red deer and Apennine chamois) and domestic (cattle, sheep, and goats) ruminants living with varying degrees of sympatry in the protected area of Maiella National Park (central Italy). According to CLSI breakpoints, 9 out of 48 isolates (18.8%) showed resistance to at least one antibiotic. One Apennine chamois isolate was resistant to all tested antibiotics. The PCR screening of related resistance genes highlighted the occurrence of *msr*C or *cfr*D in seven *Enterococcus* resistant isolates. In addition, *msr*C and *van*C genes were amplified in susceptible isolates. Specific sequences of virulence genes (*gel*E, *ace*, *efa*, *asa*1, and *esp*) related to pathogenic enterococci in humans were amplified in 21/48 isolates (43.75%), belonging mostly to wild animals (15/21; 71.42%). This is the first report of linezolid-resistant enterococci harboring virulence genes in Italian wildlife with special regard to the red deer and Apennine chamois species. The results allow us to evaluate the potential role of wild animals as indicators of antibiotic resistance in environments with different levels of anthropic pressure.

## 1. Introduction

Antimicrobial resistance (AMR) is a significant public health threat related to many factors, including the overuse or misuse of antibiotics in veterinary and human medicine. The gastrointestinal tracts of animals are considered the largest reservoir of bacteria potentially involved in the spread of antibiotic resistance [1]. Among these bacteria, the genus *Enterococcus* includes commensal Gram-positive bacteria that colonize the gastrointestinal tracts of many animal species and humans. These bacteria are important nosocomial pathogens characterized by intrinsic and acquired virulence determinants and resistance mechanisms against different antibiotics [2]. In addition, enterococci are considered responsible for several animal diseases, such as mastitis, endocarditis, diarrhea, and septicemia in bovine, pets, pigs, and poultry [3]. The pathogenicity of these bacteria is related to antibiotic resistance mechanisms and virulence factors that promote colonization in host cells and damage of tissues by means of protein and peptides [4]. Due to their adaptability to the environment and their genomic plasticity, these ubiquitous microorganisms can be employed as sentinel bacteria suitable for antibiotic resistance surveillance systems in humans, domestic animals, and wildlife. In recent years, the emergence and rapid spread of vancomycin-resistant enterococci (VRE) has resulted in the increased use of alternative molecules, such as linezolid (LNZ) and quinupristin/dalfopristin (QD), to treat VRE-related infections. These drugs are considered as the last resort in human medicine and are included in the WHO list of critically (LNZ) and highly (QD) important antibiotics [1,5]. Linezolid is an oxazolidinone that is highly effective in treating serious infections caused by multidrug-resistant Gram-positive bacteria in humans [2]. Although this molecule is not approved worldwide for use in veterinary medicine, the mobile genetic elements involved in the oxazolidinone resistance have been detected in livestock during the last few years [2,5]. Indeed, LNZ-resistant enterococci have been obtained from food-producing animals in the USA, Europe, Asia, and Africa [5,6,7,8,9,10,11]. Quinupristin/dalfopristin is a streptogramins B and streptogramins A compound that is particularly useful in treating nosocomial infections caused by resistant *E. faecium*. After the first detection in 1997, other evidence of QD-resistant isolates has been highlighted in several countries in both humans and food-producing animals [5,12]. These data suggest that the emergence of LNZ- and QD-resistant enterococci may reduce therapeutic options for the successful treatment of VRE infections in humans. Therefore, the monitoring of environmental sources (i.e., soil, water, and wild animals) that are potentially able to harbor resistant bacteria and their relative genetic elements, including pathogens hard to treat with currently available antibiotics, is considered relevant for AMR surveillance inspired by a “One Health” approach [13]. Wildlife is generally less or not at all treated with antimicrobials but may acquire resistant bacteria from the environment [14], especially where a co-existence of domestic animals, livestock, agriculture, and other human activities is widely established. In this regard, the aim of this study was to investigate the AMR against quinupristin/dalfopristin, vancomycin, and linezolid in *Enterococcus* spp. isolates from wild and domestic ungulates living in Maiella National Park (Central Italy) with varying levels of sympatry established using georeferencing data. In addition, in order to evaluate the potential pathogenicity of microorganisms under study, specific virulence genes were investigated.

## 2. Results

### 2.1. Bacterial Isolation and Antibiotic Susceptibility Test

One or more *Enterococcus* species were isolated from fecal pool samples with a total of 48 bacterial strains. In detail, 17 isolates were obtained from the group of sympatric populations (four chamois and three goats; five red deer and five sheep) and 31 from the non-sympatric animals (16 goats, 8 chamois, 7 cattle). Based on Vitek 2 analysis, 15 *E. gallinarum*, 12 *E. faecium*, 11 *E. faecalis*, 6 *E. hirae*, and 4 *E. casseliflavus* species were identified from both wild and domestic animals, as reported in Table 1. 

In greater detail, five *E. faecalis*, four *E. faecium*, three *E. gallinarum*, three *E. hirae*, and two *E. casseliflavus* were recovered from sympatric animals. Twelve *E. gallinarum,* eight *E. faecium*, six *E. faecalis*, three *E. hirae*, and two *E. casseliflavus* were isolated from non-sympatric animals.

Out of 48 isolates, 11 acquired/transferable resistant bacteria were identified, while the intrinsic resistance for QD (*E. faecalis*) and VAN (*E. casseliflavus* and *E. gallinarum*) was not considered. Nine enterococci were resistant to QD, seven to LNZ, and one to VAN. In detail, phenotypic resistance isolates were detected in three wild sympatric, two domestic sympatric, and four domestic non-sympatric animals. Only one *E. faecium* isolate, detected from an Apennine chamois belonging to the sympatric animal group, showed multiple resistance to QD, LNZ, and VAN.

### 2.2. Antibiotic Resistance Genes and Virulence Determinants

Among the QD resistance genes investigated, the *msr*C gene was detected in 20/48 (41.66%) isolates, including one QD intrinsically resistant isolate and six QD acquired/transferable resistant bacteria. The remaining 13 enterococci results were considered sensitive to QD by the antibiotic susceptibility analysis. The VAN resistance genes were not amplified, except for the *van*C1 and *van*C2 observed in nine intrinsically resistant enterococci and in one sensitive isolate as reported in Table 1. All enterococci under study were negative for the LNZ resistance genes *optr*A, *cfr, cfr*(B), and *poxt*A, but the *cfr*(D) gene was amplified in all the LNZ-resistant isolates. 

In 21/48 (43.75%) isolates, the virulence gene fragments under study were amplified, except for the *hyl* gene. The most representative gene was *gel*E (17/48, 35.41%), followed by *asa*1 (12/48, 25%), *efa* (11/48, 22.91%), *ace* (4/48, 0.08%), and *esp* (2/48, 0.04%). In detail, *gel*E and *efa* genes were predominant in wild animals (94.11% and 81.81%, respectively), and all four *ace*-positive isolates were obtained from Apennine chamois populations (Table 1). Finally, the *asa*1 fragment was amplified in both wild and domestic animals. Co- occurrence of virulence genes was observed in several isolates. The *gel*E/*efa* and *gel*E/*asa*1 genes were observed in three isolates, while eight isolates showed multiple positivity for *gel*E/*efa*/*asa*1, *gel*E/*efa*/*esp*, *gel*E/*efa*/*ace*, or *esp*/*efa*/*asa*1, along with two isolates being positive for *gel*E/*efa*/*ace*/*asa*1. Table 1 shows the details of gene occurrence in the samples under study.

For both antibiotic resistance and virulence genes, the analysis of amplicons confirmed the specificity of PCR reactions, showing an identity between 98–99% (coverage 93–96%) with homologous sequences deposited in GenBank.

## 3. Discussion

In this study, the resistance against critically and highly important antibiotics in enterococci isolated from wild and domestic ruminants was tested in order to provide new information about the spread of resistance to these antibiotics in the environment. Among them, VAN was selected for the relevance of VRE in human health, while LNZ and QD were investigated because they are considered alternative molecules to treat VRE-infections. 

In Europe, several authors have tested enterococci recovered from free-ranging terrestrial wild mammals against different antibiotics. Isolates from chamois have shown resistant results to tetracyclines, erythromycin, and VAN in Poland and Slovakia [15,16]. During the last few years in Spain and Portugal, the enterococci obtained from carnivores, ungulates, and wild rabbits have shown a phenotypic or genetic resistance against QD and LNZ in wild boars and Iberian wolves; VAN in rabbits, wild boars, and roe deer; and both QD and VAN in foxes [17,18,19,20,21,22,23,24]. A similar investigation was carried out in Italy (Tuscany region) involving different species, but only VAN resistance in three enterococci sourced from a wolf, a mouflon, and a wild boar was observed [14]. Compared to the aforementioned papers, the present study described for the first time enterococci resistant to LNZ and QD in Italian free-ranging Apennine chamois and red deer.

LNZ resistance was only described in one isolate from sympatric Apennine chamois, two isolates from sympatric red deer, and four bacteria from non-sympatric goats. While LNZ is not licensed for food-producing animals, LNZ-resistant isolates have been reported in European countries in domestic animals, and recent data in wildlife have aroused interest on environmental contamination [6,7,8,9,10,11,22]. Furthermore, molecular investigations have allowed the detection of the LNZ resistance gene, *cfr*(D), in all phenotypical resistant *E. faecium* and *E. gallinarum*. This gene has recently been described as a new variant of the *cfr* gene in *E. faecium* from human clinical isolates in France, Ireland, the Netherlands, and Australia [25,26,27,28], and in *E. faecalis* from Spanish hospital isolates [11]. 

The detection of VAN resistance genes was mostly related to intrinsically resistant isolates, but, interestingly, a positive result was obtained from a sensitive *E. faecium*, while in the resistant *E. faecium* none of the investigated VAN genes was detected. Other factors may be involved in the expression of genetic information or in the phenotypic resistance. As suggested by other authors, these factors may be related to environmental conditions and may modify the expression of a specific gene and the phenotypic profile. Specifically, the modulation role of collective resistance was described, including mechanisms where the phenotypic behavior of a bacterium was modified by communities of bacteria (i.e., biofilm formation and indirect resistance). Additionally, a low, host-related growth rate or the presence of specific metabolites, such as oxygens radicals, may have modified resistance [29]. 

The *msr*C gene was amplified in both QD-resistant and sensitive isolates, suggesting that this gene may be silent or involved in resistance to other antibiotics not tested in this study. Indeed, this gene has been related to erythromycin-resistant enterococci derived from domestic animals and different wild mammals [5,14]. 

Furthermore, the patterns of resistance observed in this study showed some differences in relation to the settings and the populations sampled. The resistant isolates were found in sympatric wild and domestic animals (Apennine chamois and goats; red deer and sheep) and in non-sympatric livestock (goats), rather than in non-sympatric Apennine chamois living in territories where human activities are limited and extensive livestock is not admitted. This evidence suggests the potential role of interactions with livestock and a shared environment as sources of antibiotic resistance determinants for wildlife: using the same land may provide an opportunity to share the resistome. 

The virulence genes detected in this study are relevant in nosocomial infections and they have a direct effect on host colonization and immune response escapement [30]. The encoding gene of the aggregation substance (*gel*E) was detected mostly in resistant bacteria from red deer and, along with the genes encoding the endocarditis antigen (*efa*), the collagen-binding protein (*ace*), and the aggregation substance (*asa*1), in isolates from Apennine chamois. The *gel*E gene appears to increase the biofilm formation or aggregation, and it was previously detected in wild animals in Poland, along with the *efa* gene that provides a similar function [31]. In Italy, the detection of *gel*E, *ace*, and *asa*1 was reported in different species of wild mammals [14]. Interestingly, isolates with three or four virulence genes found in this study were mainly distributed in Apennine chamois samples. 

In detail, out of twelve isolates from sympatric and non-sympatric Apennine chamois, eleven bacteria showed occurrence of virulence genes. As suggested from other authors, these multi-virulent profiles may be related to the adaptation of the hosts rather than to an increase in virulence [31]. However, some strains harboring virulence factors also showed resistance to highly or critically important antibiotics, such as two isolates from Apennine chamois, enhancing their potential pathogenic roles. In addition, the virulence factors found in *E. gallinarum*, *E. casseliflavus*, and *E. hirae* species have been poorly investigated in previous studies [4].

In the future, a whole-genome analysis of these isolates could improve the evaluation of the relationships among the bacteria, environmental sources, and animals investigated. Indeed, the coexistence in commensal enterococci of resistant profiles against medically important antibiotics, along with several virulence genes, highlights the need to investigate the impact of human activities and, in particular, the food-producing livestock industry on the environment and its role as a potential source of AMR determinants [13,32].

## 4. Materials and Methods

### 4.1. Study Area and Sampling Design

**Figure 1:**
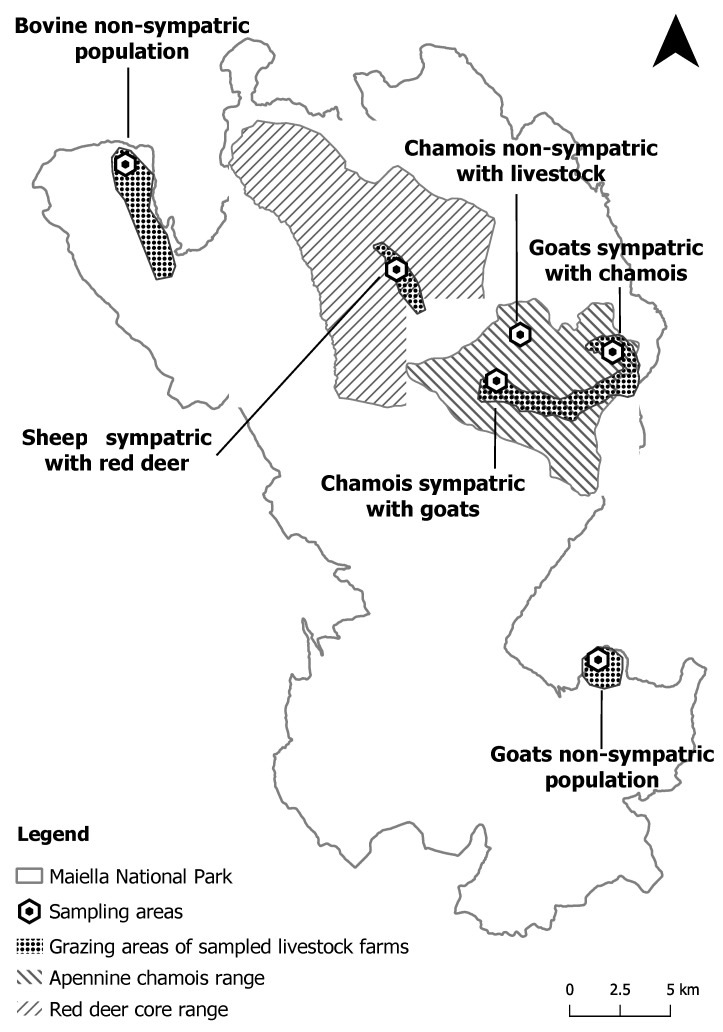
Maiella National Park boundaries, geographic distribution of sympatric and non-sympatric animals, and sampling areas.

The Maiella National Park (MNP) covers a vast, mountainous area of about 740 km^2^ in the central Apennine Mountains and is home to several diversified vertebrate fauna, including mammalian species relevant at national and international levels listed in the Habitats Directive (92/43/EEC). Among them, the Apennine chamois (*Rupicapra pyrenaicaornata*) is a rare subspecies of chamois living in extremely limited areas of central Italy, including the territories of MNP, where the current population size is approximately 1300 individuals as a result of a reintroduction program carried out in the past. This species coexists with more widespread wild ungulates, such as red deer (*Cervus elaphus*, approximately 1500 individuals), and domestic livestock (cattle, sheep, and goats) traditionally raised on small farms with extensive grazing systems. In this regard, the distribution areas of wild and domestic animals were previously determined by georeferencing data in order to define the level of grazing land sharing. The populations under study are routinely monitored by the MNP technical staff. The Apennine chamois population (size and distribution area) is defined using a census block technique, consistently performed in summer and autumn over the last twenty years. The information regarding demographic structure and territory occupancy of the main herds is obtained by GPS collars, ear tags, and direct visual observations that have been conducted in a systematic design during summer and autumn of the last ten years. The red deer population is defined by estimating the minimum number of reproductive males during the rutting season and combining this information with data on population demographic structure that is established by recurrent visual observations during all the seasons. Additionally, the farms of domestic animals require a specific authorization every year to use the grazing lands, and each livestock group is assigned an area defined by GPS coordinates. Based on that, the first group of animals, including sympatric populations (100 Apennine chamois coexisting with a farm of 120 goats, and 50 red deer with a farm of 300 sheep), and the second group of non-sympatric populations (70 cattle, 210 goats, and 100 Apennine chamois) were each distributed in different areas of the MNP (Figure 1).

From October to November 2019, fecal samples from red deer, Apennine chamois, and domestic ruminants were collected. In order to obtain fresh feces suitable for microbiological investigations, the different groups of grazing animals were followed and observed with no disturbance to their physiological activities, and the fecal samples were collected while limiting as much as possible any contamination with the soil. Based on the sampling design, a total of 132 fecal samples were recovered and considered suitable for laboratory analysis. Subsequently, the specimens were grouped in 33 fecal pools with 4 fecal samples for each pool (Table 2) and were stored at 4 °C and analyzed within 24 h after the collection.

### 4.2. Bacteria Isolation and Antibioticssusceptibility Test

*Enterococcus* spp. isolates were obtained by a preliminary non-selective enrichment of fecal samples in buffered peptone water (24 h at 37 °C), followed by subculturing on Slanetz and Bartley agar (Liofilchem, Italy) at 37 °C for 48 h. From each plate, 1 or 2 representative colonies, morphologically referred to *Enterococcus* genus and adequately isolated from other microorganisms, were selected to obtain pure subcultures. The species identification of typical colonies and the antimicrobial susceptibility tests for QD, VAN, and LNZ were performed using a Vitek 2 system (Biomerieux, France) following the instructions given by the manufacturer. The minimum inhibitory concentration (MIC) values were evaluated based on the Clinical and Laboratory Standards Institute (CLSI) breakpoints considered relevant for human health [33].

### 4.3. Detection of Antibiotic Resistance and Virulence Genes

The genes related to QD (*vg*A, *msr*C, *Vat*D, *vgb*B, *vgb*A, *erm*B, and *vat*E), VAN (*van*A, *van*B, *van*C1, *van*C2, *van*D, *van*M, and *van*N), and LNZ (*cfr*, *cfr*(B), *cfr*(D), *optr*A, and *poxt*A) resistance and those encoding some virulence factors (*gel*E, *esp*, *efa*, *ace*, *hyl*, and *asa*1) in enterococci were amplified by previously described protocols of end-point PCR (Table 3). The amplicons with the expected size were purified, sequenced, and compared with those included in the GenBank database using Chromas software, FASTA (http://www.ebi.ac.uk/fasta33, accessed on 20 January 2022), Clustal Omega (http://www.ebi.ac.uk/Tools/msa/clustalo, accessed on 20 January 2022), and the Basic Local Alignment Search Tool (BLAST) to confirm the specificity of protocols applied. The sequences obtained were submitted to the GenBank database with accession numbers from OM525845 to OM525851.

## 5. Conclusions

In conclusion, the combined analysis of laboratory and georeferenced data applied in this study provided new information about emerging resistance patterns in commensal and potentially pathogenic enterococci and suggests the importance of a multidisciplinary approach to the AMR challenge. The wild species investigated in this study were strictly linked to the territory of the MNP, which is characterized by complex ecosystems with different levels of human–animal–environment interactions. Therefore, they may be considered sentinel species of emerging antibiotic resistance patterns. Additional studies should include both human and ungulate-derived isolates in order to assess the relationships and to confirm the importance of animal–human interactions in the transmission of antibiotic resistance organisms.

## Figures and Tables

**Table 1:** Details of bacterial species, animal sources, MIC values, and genes detected in this study.

Groups	Strain	Animal Source	MIC (μg/mL) *			Genes	
			QD	VAN	LNZ	Resistance	Virulence
**Sympatric populations**	*E. faecalis*	Apennine Chamois	4	1	2		*gel*E, *efa*, *asa*1
	*E. faecalis*	Apennine Chamois	4	1	2		*gel*E, *efa*, *asa*1
	*E. faecalis*	Goats	4	1	2	*van*C1/C2, *msr*C	*gel*E, *efa*, *asa*1
	*E. hirae*	Apennine Chamois	1	4	2		*gel*E
	*E. hirae*	Goats	1	0.5	2		
	*E. faecium*	Apennine Chamois	**16**	**32**	**8**	*msr*C, *cfr*D	*gel*E, *efa*
	*E. casselliflavus*	Goats	1	4	2	*van*C1/C2	
	*E. faecalis*	Red deer	4	1	2		*gel*E
	*E. faecalis*	Red deer	4	1	2		*gel*E
	*E. faecium*	Red deer	**4**	1	2	*msr*C	*gel*E
	*E. faecium*	Red deer	0.5	0.5	2		
	*E. faecium*	Sheep	1	0.5	2	*msr*C	
	*E. gallinarum*	Red deer	**16**	32	**8**	*van*C1, *msr*C, *cfr*D	*gel*E
	*E. gallinarum*	Sheep	**16**	32	**8**	*van*C1/C2, *cfr*D	
	*E. gallinarum*	Sheep	**16**	32	**8**	*van*C1, *msr*C, *cfr*D	*gel*E, *esp*, *efa*
	*E. hirae*	Sheep	1	0.5	2		
	*E. casselliflavus*	Sheep	1	4	1		
**Non-sympatric populations**	*E. gallinarum*	Cattle	1	2	0.5		
	*E. casseliflavus*	Cattle	1	1	2	*van*C2	
	*E. faecium*	Cattle	1	2	1	*msr*C	
	*E. faecium*	Cattle	1	2	0.5	*msr*C	
	*E. faecium*	Cattle	1	2	0.5	*msr*C	
	*E. faecium*	Cattle	1	2	0.5	*msr*C	
	*E. faecium*	Cattle	1	2	0.5	*msr*C	
	*E. gallinarum*	Goats	1	2	0.5		
	*E. gallinarum*	Goats	**16**	8	**32**	*msr*C, *van*C1/C2, *cfr*D	
	*E. gallinarum*	Goats	**16**	8	**32**	*msr*C, *cfr*D	
	*E. gallinarum*	Goats	0.5	2	0.5	*msr*C	
	*E. gallinarum*	Goats	1	2	0.5		
	*E. gallinarum*	Goats	1	2	0.5	*van*C1/C2, *msr*C	*asa*1
	*E. gallinarum*	Goats	1	2	0.5	*msr*C	
	*E. gallinarum*	Goats	0.5	0.5	2	*msr*C	
	*E. gallinarum*	Goats	**4**	1	2	*msr*C	*esp, efa, asa*1
	*E. gallinarum*	Goats	**16**	32	**8**	*van*C2, *cfr*D	
	*E. gallinarum*	Goats	1	2	0.5		
	*E. faecium*	Goats	0.5	2	0.5	*msr*C	
	*E. faecium*	Goats	0.5	2	4	*msr*C	
	*E. faecium*	Goats	0.5	2	0.5		
	*E. hirae*	Goats	1	2	0.5		*asa*1
	*E. hirae*	Goats	1	2	0.5		*asa*1
	*E. faecalis*	Apennine Chamois	2	2	1		*gel*E, *asa*1
	*E. faecalis*	Apennine Chamois	2	2	1		*gel*E, *efa*, *ace*
	*E. faecalis*	Apennine Chamois	4	2	1		*gel*E, *efa*, *ace*
	*E. faecalis*	Apennine Chamois	4	2	1		*gel*E, *asa*1
	*E. faecalis*	Apennine Chamois	4	2	1		*gel*E, *efa*, *ace*, *asa*1
	*E. faecalis*	Apennine Chamois	4	2	1		*gel*E, *efa*, *asa*1
	*E. casseliflavus*	Apennine Chamois	1	2	4	*van*C2	*gel*E, *efa*, *ace*, *asa*1
	*E. hirae*	Apennine Chamois	1	2	0.5		

* QD: quinupristin/dalfopristin; VAN: vancomycin; LNZ: linezolid. The MIC values related to the resistance based on the CLSI breakpoints are indicated in bold.

**Table 2:** Number of fecal pools and colonies selected for testing from each population under study. The number of collected samples for each population is reported in parentheses.

Groups	Animals	N. Fecal Pools	N. Colonies
**Sympatric populations**	Apennine chamois (8)	2	4
	Goat (12)	3	3
	Red deer (16)	4	5
	Sheep (12)	3	5
**Non-sympatric populations**	Cattle (20)	5	7
	Goat (32)	8	16
	Red deer (12)	3	0
	Apennine chamois (20)	5	8
**Total**		33	48

**Table 3:** Details of single and multiplex PCR protocols applied for antibiotic resistance and virulence gene detection.

Primer	Sequence 5’- 3’	Size (bp)	References
VanD_F1	TGGAATCACAAAATCCGGCG	311	[34]
VanD_R2	TWCCCGCATTTTTCACAACS		
VanM_F1	GGCAGAGATTGCCAACAACA	425	
VanM _R1	AGGTAAACGAATCTGCCGCT		
VanC2_F1	GCAAACGTTGGTACCTGATG	523	
VanC2_R4	GGTGATTTTGGCGCTGATCA		
VanB_F1	GATGTGTCGGTAAAATCCGC	640	
VanB_R1	CCACTTCGCCGACAATCAAA		
VanA_F1	GCAAGTCAGGTGAAGATGGA	721	
VanA_R1	GCTAATACGATCAAGCGGTC		
VanC1_5	GTATCAAGGAAACCTCGCGA	836	
VanC1_6	CGTAGGATAACCCGACTTCC		
VanN_F1	CCTCAAATCAGCAGCTAGTG	941	
VanN_R1	GCTCCTGATAAGTGATACCC		
Cfr_fw	TGAAGTATAAAGCAGGTTGGGAGTCAAC	746	[35]
Cfr_rev	CATATAATTGACCACAAGCAGC		
optrA_fw	TACTTGATGAACCTACTAACCA	422	
optrA_rev	CCTTGAACTACTGATTCTCGG		
poxtAfw	AAAGCTACCCATAAAATATC	533	
poxtArev	TCATCAAGCTGTTCGAGTTC		
cfr(B) fw	TGAGCATATACGAGTAACCTCAAGA	293	[36]
cfr(B) rev	CGCAAGCAGCGTCTATATCA		
cfr(D) fw	AGAAGTCGCAACAAGTGAGGA	595	[11]
cfr(D) rev	GCAACTGCATGAGTCAAAGAA		
Vat D F	TCCAGCTAACATGTATGGCG	271	[37]
Vat D R	GCTCAATAGGACCAGGTGTA		
vgaA F	AGTGGTGGTGAAGTAACACG	659	
vgaA R	CTTGTCTCCTCCGCGAATAC		
vgaB F	TGACAATATGAGTGGTGGTG	576	
vgaB R	GCGACCATGAAATTGCTCTC		
vgbB F	CAGCAGTCTAGATCAGAGTGG	728	[37]
vgbB R	CATACGGATCCATCTTTTCC		
msrC F	AAGGAATCCTTCTCTCTCCG	343	
msrC R	GTAAACAAAATCGTTCCCG		
vgbA F	TACAGAGTACCCACTACCGA	569	
vgbA R	TCAATTCCTGCTCCAGCAGT		
ermB F	CATTTAACGACGAAACTGGC	424	
ermB R	GGAACATCTGTGGTATGGCG		
vatE F	ACTATACCTGACGCAAATGC	511	[37]
vatE R	GGTTCAAATCTTGGTCCG		
gelE F	TATGACAATGCTTTTTGGGAT	213	[38]
gelE R	AGATGCACCCGAAATAATATA		
esp F	AGATTTCATCTTTGATTCTTGG	510	
esp R	AATTGATTCTTTAGCATCTGG		
ace F	GAATTGAGCAAAAGTTCAATCG	1008	
ace R	GTCTGTCTTTTCACTTGTTTC		
efa F	GCCAATTGGGACAGACCCTC	688	
efa R	CGCCTTCTGTTCCTTCTTTGGC		
asa1 F	GCACGCTATTACGAACTATGA	375	[38]
asa1 R	TAAGAAAGAACATCACCACGA		
hyl F	ACAGAAGAGCTGCAGGAAATG	276	
hyl R	GACTGACGTCCAAGTTTCCAA		
cylA F	ACTCGGGGATTGATAGGC	688	
cylA R	GCTGCTAAAGCTGCGCTT		

## Data Availability

Data will be made available upon reasonable request to the corresponding author.
